# Vitronectin and Its Interaction with PAI-1 Suggests a Functional Link to Vascular Changes in AMD Pathobiology

**DOI:** 10.3390/cells11111766

**Published:** 2022-05-27

**Authors:** Fabiola Biasella, Tobias Strunz, Christina Kiel, on behalf of the International AMD Genomics Consortium (IAMDGC), Bernhard H. F. Weber, Ulrike Friedrich

**Affiliations:** 1 Institute of Human Genetics, University of Regensburg, Franz-Josef-Strauss-Allee 11, 93053 Regensburg, Germany; fabiola.biasella@klinik.uni-regensburg.de (F.B.); tobias.strunz@klinik.uni-regensburg.de (T.S.); christina.kiel@klinik.uni-regensburg.de (C.K.); 2 John P. Hussman Institute for Human Genomics, University of Miami, Miami, FL 33136, USA; sec@miamiedu.onmicrosoft.com; 3 Institute of Clinical Human Genetics, University Hospital Regensburg, Franz-Josef-Strauss-Allee 11, 93053 Regensburg, Germany

**Keywords:** AMD, age-related macular degeneration, vitronectin, VTN, rs704, SERPINE1, PAI-1, neovascularization

## Abstract

The pathogenesis of age-related macular degeneration (AMD), a frequent disorder of the central retina, is incompletely understood. Genome-wide association studies (GWAS) suggest a strong contribution of genomic variation in AMD susceptibility. Nevertheless, little is known about biological mechanisms of the disease. We reported previously that the AMD-associated polymorphism rs704C > T in the vitronectin (*VTN*) gene influences protein expression and functional aspects of encoded vitronectin, a human blood and extracellular matrix (ECM) protein. Here, we refined the association of rs704 with AMD in 16,144 cases and 17,832 controls and noted that rs704 is carried exclusively by the neovascular AMD subtype. Interaction studies demonstrate that rs704 affects the ability of vitronectin to bind the angiogenic regulator plasminogen activator inhibitor 1 (PAI-1) but has no influence on stabilizing its active state. Western blot analysis and confocal imaging reveal a strong enrichment of PAI-1 in the ECM of cultured endothelial cells and RPE cell line ARPE-19 exposed to vitronectin. Large-scale gene expression of *VTN* and *PAI-1* showed positive correlations and a statistically significant increase in human retinal and blood tissues aged 60 years and older. Our results suggest a mechanism by which the AMD-associated rs704 variant in combination with ageing may contribute to the vascular complications in AMD.

## 1. Introduction

Age-related macular degeneration (AMD) is a common progressive disorder of the central retina that irreversibly impairs visual acuity and field of vision [1,2]. Oxidative stress associated with mitochondrial dysfunction, dysregulated immune activation, extracellular matrix (ECM) alterations, and choroidal vascular disturbances are discussed to be part of the pathological mechanisms involved in disease development and progression [2]. Early stages of the disease are characterized by the formation of abnormal insoluble extracellular deposits (drusen and subretinal drusenoid deposits) and retinal pigment epithelium (RPE) malfunction in the macular area, which appears to be an initial insult to the integrity of the neuro-retinal layers. In the late stages of AMD, the retina presents with severe structural and functional lesions characterized by geographic atrophy (GA) and/or neovascular (NV) complications [2]. A combination of a number of factors including age, genetic background, lifestyle, and environmental conditions influence personal susceptibility to AMD [3]. Genome-wide association studies (GWAS) revealed a strong genetic contribution to AMD by identifying genetic loci and pathways associated with disease at genome-wide significance. Consequently, these studies provide an excellent framework for further functional research aimed at understanding the molecular and cellular mechanisms underlying AMD pathobiology.

Genetic variant rs704, a common non-synonymous polymorphism (Thr400Met) in the human vitronectin (*VTN*) gene, is strongly associated with AMD. Specifically, rs704 is part of a 95% credible set of 22 genetic variants at the AMD-associated *TMEM97*-*VTN* locus on chromosome 17 (17q11.2) [4]. The vitronectin protein is a secreted glycoprotein abundant in plasma and in the ECM of various tissues, including the retina, where this protein has been detected mainly at the choroid/Bruch’s membrane/RPE interface [5,6,7]. Interactions with lipids, glycosaminoglycans, collagens, plasminogen activator inhibitor-1 (PAI-1) and adhesion receptors make vitronectin a multifunctional protein involved in the regulation of a variety of cellular events that are also known to be affected by AMD, such as ECM deposition, cell adhesion and migration, immune response, angiogenesis and fibrinolysis [8,9,10,11,12,13,14,15]. Notably, vitronectin has repeatedly been identified as the main component and coordinating factor in the formation of AMD-associated retinal deposits [16,17,18,19].

We have recently shown that the AMD-associated polymorphism rs704 influences vitronectin protein expression, endoproteolytic processing, and vitronectin-regulated cellular events such as ECM deposition and cell migration [8]. The present study now aims to further deepen our understanding of the contribution of the two vitronectin isoforms and their interaction partner PAI-1 to AMD pathology. Our data suggest that a rs704-modified interaction between vitronectin and PAI-1 could contribute to AMD-related vascular changes.

## 2. Material and Methods

### 2.1. Description of Datasets

The IAMDGC dataset comprises genotype and phenotype information on 16,144 late-stage AMD patients and 17,832 control individuals. The AMD phenotypes are categorized into three groups, including 10,749 NV-AMD, 3235 individuals with the GA-AMD, and 2160 individuals with combined NV/GA disease. A total of 19,624 women and 14,352 males of European descent were included in the study. Furthermore, the IAMDGC study comprised an additional 6657 patient data with early or intermediate AMD. Detailed information on selection criteria, clinical phenotyping, quality control, and genetic imputation are given in Ref. [4].

The Genotype-Tissue Expression (GTEx) consortium version 8 data were used to investigate gene expression in whole blood samples [20]. GTEx data processing protocols and filtering for European individuals are detailed in Ref. [21]. Overall, 556 whole blood samples were analyzed. Data not provided through the GTEx web portal, such as covariates and age of donors, were downloaded from dbGaP (accession ID: phs000424.v8.p2). A dataset analyzing gene expression in 311 healthy retinal tissue samples from three independent studies [22] was available for age-dependent gene expression analysis in retina.

### 2.2. Association Analysis

Association analysis of rs704 with AMD subtypes was performed based on a logistic regression model provided by the *glm* function in R [23]. Depending on the analysis, the model was adjusted for age, gender, the source of DNA, and the first two genotype principal components. The false discovery rate (FDR, Q-value) [24] was applied to correct for multiple testing using the multi-test package [25]. Gender-specific association was calculated by the same method, with the difference that the association was determined only in individuals of the respective sex and gender was removed as a covariate. To analyze subtype-specific associations of the loci harboring the genes *VTN* (ENSG00000109072) and *PAI-1* (aka *SERPINE1*, ENSG00000106366), associations were calculated as described above for the regions of two mega-base pairs (Mbp) each (*VTN* locus: chromosome 17; 25,649,724–27,649,724 and *PAI-1* locus: chromosome 7; 99,735,887–101,735,887; GRCh37). Subtype-specific association results were visualized by generating locuszoom plots [26,27] representing an 0.8 Mbp region around the association peak.

### 2.3. Linear Regression Analysis of Gene Expression

Analysis of gene expression correlation was performed in Ref. [23] using the *lm* function. Grouping by age was achieved with the cut function, which is part of the R core distribution.

### 2.4. Cell Culture

Cultivation of HEK293-EBNA (Invitrogen, Waltham, MA, USA), HUVECs (Life Technologies, Carlsbad, CA, USA) and ARPE-19 cells (American Type Culture Collection, Manassas, VA, USA) was performed as described in Ref. [8] and includes descriptions of media and media supplements.

### 2.5. RNA Isolation and Reverse Transcription (RT) into Complementary DNA (cDNA)

RNA isolation, cDNA synthesis, kits and enzymes are given in Ref. [8].

### 2.6. Quantitative Real-Time Polymerase Chain Reaction (qRT-PCR)

Takyon ™ Low Rox Probe MasterMix dTTP blue (Eurogentec, Seraing, Liège, Belgium) and KiCqStart ^®^ Probe Assay (Sigma-Aldrich, St. Louis, MO, USA) were applied for gene expression analysis by qRT-PCR. DNA polymerase, oligonucleotide primer sequences and probe names are given in Supplementary Table S1. Each reaction was performed in technical triplicates using the QuantStudio ™ 5 Real-Time PCR System (Thermo Fisher Scientific, Waltham, MA, USA). Data were analyzed applying the ΔΔCt method for relative quantification [28].

### 2.7. Expression Constructs

The coding sequence for PAI-1 (NM_000602.4) was amplified from cDNA of ARPE-19 cells. The fragment was cloned into the EcoRI/XhoI site of the pEXPR-IBA103 vector (IBA Life Sciences, Göttingen, Germany), and thus fused to a Twin-Strep-tag (IBA Life Sciences) in a generated vector named pEXPR-IBA103-PAI-1. Cloned products were verified by Sanger sequencing of plasmid DNA using the BigDye Terminator v1.1 Cycle Sequencing Kit (Thermo Fisher). Oligonucleotide primer sequences and specifics of the DNA polymerase used are given in Supplementary Table S1.

Generation of expression constructs used for heterologous expression and purification of the VTN (NM_000638.3) isoforms (Strep-tagged vitronectin isoforms: pEXPR-IBA103-VTN_rs704: C or pEXPR-IBA103-VTN_rs704: T; untagged vitronectin isoforms: pcDNA3.1-VTN_rs704: C or pcDNA3.1-VTN_rs704: T) is described in [8]. All expression constructs were transfected in HEK293-EBNA applying the calcium phosphate method [29].

### 2.8. Purification of the Recombinant Proteins PAI-1 and Vitronectin

Isolation and purification of Strep-tagged recombinant PAI-1 and vitronectin isoforms VTN_rs704: C and VTN_rs704: T from the cultivation medium of HEK293-EBNA transfected with pEXPR-IBA103-PAI-1, pEXPR-IBA103-VTN_rs704: C or pEXPR-IBA103-VTN_rs704: T was carried out using Strep-tag protein purification technology (IBA Life Sciences), as described in [8]. The cultivation medium of HEK293-EBNA transfected with an empty pEXPR-IBA103 vector was subjected to the same purification procedure, and eluates were used as the control (“control eluate”). Purification was visually inspected by Coomassie blue staining and Western blot analysis (Supplementary Figure S1). Strep-tagged PAI-1 was recognized as a major molecular weight species slightly above 55 kDa, with an occasionally appearing additional lower molecular weight, as reported earlier for purified PAI-1 [30,31]. Purified strep-tagged VTN_rs704: C and VTN_rs704: T showed stains with a molecular weight of about 75 kDa and 65 kDa, as previously described [8,32].

Concentrations of purified proteins were measured with the RotiQuant reagents (Carl Roth GmbH, Karlsruhe, Germany) according to the method of Bradford [33]. Bovine serum albumin (BSA; AppliChem GmbH, Darmstadt, Germany) served as the standard.

### 2.9. SDS-PAGE and Western Blot Analysis

SDS-PAGE and Western blot analysis were conducted as described [34,35]. Densitometric quantification of Western blots was performed with the Image Studio software (LI-COR Biosciences, Lincoln, NE, USA).

### 2.10. Antibodies

Antibodies, their origin and dilutions applied in Western blot or immunofluorescence assays are given in Supplementary Table S2.

### 2.11. Affinity Chromatography of Vitronectin Isoforms to Immobilized PAI-1

Binding of vitronectin isoforms VTN_rs704:C and VTN_rs704: T to PAI-1 was investigated by affinity chromatography of vitronectin to immobilized PAI-1. Briefly, 75 µL of purified recombinant Strep-tagged PAI-1 (100 µg/mL) was loaded onto gravity flow columns (0.2 mL Strep-Tactin Sepharose columns; IBA Life Sciences) pre-equilibrated with 200 µL of wash buffer (Buffer W; IBA Life Sciences). To allow PAI-1 immobilization, columns were incubated with Strep-tagged PAI-1 for 15 min. After washing the columns five times with 200 µL wash buffer, 2.0 mL of medium containing untagged VTN_rs704: C or VTN_rs704: T (FCS-free supernatant of HEK293-EBNA cells transfected with pcDNA3.1-VTN_rs704: C or pcDNA3.1-VTN_rs704: T, adjusted for vitronectin concentrations) was loaded onto the columns and incubated for 1 h. The concentration of untagged recombinant vitronectin was estimated by comparative Western blot analysis with a commercially available recombinant vitronectin protein (10424-H08H; Sino Biological, Inc., Beijing, China).

After six washing steps with 200 µL wash buffer (Supplementary Figure S2A), proteins were eluted five times with 100 µL elution buffer (Buffer E; IBA Life Sciences). Pooled elution fractions were loaded on SDS-PAGE and co-precipitated proteins were subjected to Western blot analysis.

To investigate putative non-specific binding of vitronectin to the column material, the VTN_rs704: C- or VTN_rs704: T-containing medium was loaded onto PAI-1-free columns in otherwise identical affinity chromatography experimental procedures (Supplementary Figure S2A–C).

### 2.12. PAI-1 Activity Assay

The effects of vitronectin isoforms VTN_rs704: C and VTN_rs704: T on PAI-1 activity were followed by incubating 7.5 µg/mL of purified recombinant PAI-1 with 7.5 µg/mL of purified recombinant VTN_rs704: C, VTN_rs704: T, or control eluate in elution buffer for 7 h at 37 °C. After time points of 0, 1, 2, 3, 4, 5, and 7 h, 10 µL aliquots were removed and used to test the vitronectin-dependent PAI-1 activity. To this end, samples were subjected to an enzymatic assay measuring the capacity of PAI-1 to inhibit its physiological ligand urokinase plasminogen activator (uPA) [36]. The inhibition of uPA activity by PAI-1 was colorimetrically assessed via the quantification of a chromogenic substrate cleaved from active uPA using the CHEMICON PAI Activity Assay Kit (Sigma-Aldrich), according to the manufacturer’s protocol. This required an additional incubation step, first of preincubated vitronectin/control eluate and PAI-1-containing samples with uPA for 30 min, followed by the chromogenic enzymatic reaction for 2 h. Subsequently, absorbance of the chromogenic substrate was measured at 405 nm using a Spark multimode microplate reader (Tecan Group AG, Männedorf, Switzerland).

### 2.13. Endogenous PAI-1 Expression and Secretion by Vascular Endothelial Cells after Exposure to Recombinant Vitronectin Isoforms

Confluent HUVECs (passages 3–4) from a T-25 flask were rinsed with Dulbecco’s phosphate-buffered saline (DPBS; Sigma-Aldrich) and incubated with 1 mL of Gibco Trypsin–EDTA (Thermo Fisher Scientific) for 1 min at room temperature. After stopping Trypsin reaction with 20% FCS in DPBS, cells were centrifuged for 5 min at 1000 rpm. To remove traces of FCS, cell pellets were washed once in DPBS and centrifuged as before. Cells were finally resuspended in FCS-free medium and seeded onto 24-well plates at a density of 3.75 × 10^5^ cells/well. After seeding, 20 µg/mL of purified recombinant VTN_rs704: C, VTN_rs704: T, or control eluate was added to the cells, and incubated for 24 h at 37 °C. Afterwards, medium was removed and HUVECs were gently scraped with a mini cell scraper (VWR International) in 300 µL DPBS. Cell suspension was centrifuged for 5 min at 4600 rpm, the supernatant was discarded, and the cell pellet was resuspended in 50 µL DPBS. To harvest HUVEC-deposited ECM, 200 µL of PBS containing 0.5% Triton X-100 and 20 mM NH_4_OH were added to the decellularized wells for 5 min at room temperature to lyse any remaining cells. After washing 5 times with DPBS, 40 µL of hot (95 °C) Laemmli buffer mixed with 100 mM dithiothreitol (DTT) was pipetted onto the wells and the ECM was scraped off. Harvested cells and ECM samples were subjected to SDS-PAGE followed by Western blot analysis. Endogenous PAI-1 was detected as a major molecular weight species of approximately 50 kDa, which was quantified by densitometric analysis.

To assess PAI-1 mRNA expression 5.0 × 10^5^ HUVECs/well were seeded and treated as described above. After RNA isolation, the expression of *PAI-1* and the housekeeping gene *HPRT* as a control was followed by qRT-PCR.

### 2.14. Analysis of Vitronectin-Dependent Deposition of Endogenous PAI-1 in ARPE19-Derived ECM

The influence of vitronectin isoforms on endogenous PAI-1 deposition into the ECM was investigated by immunolabeling of ECM deposited from ARPE-19 cells heterologously expressing VTN_rs704: C, VTN_rs704: T, or empty pcDNA3.1 vector (Invitrogen) as a control. Cell transfection, cultivation, and ECM preparation were conducted as described in [8], but with an extended cultivation time of 8 weeks. In short, ARPE-19 cells were transfected with pcDNA3.1-VTN_rs704: C, pcDNA3.1-VTN_rs704: T, or empty pcDNA3.1. After 24 h, cells were seeded onto 12-well transwell inserts (0.4-µm pore size; Greiner Bio-One GmbH, Frickenhausen, Germany). Confluent monolayers were cultured in ARPE-19 cultivation medium deprived of FCS and supplemented with dextran sulphate and ascorbic acid (crowded conditions), as described in [37]. After 8 weeks, transwell filter inserts were decellularized and immunofluorescent labelling of the deposited ECM was performed as described in [38]. Confocal microscopy imaging and image evaluations were conducted as described [8].

### 2.15. Statistical Analysis

Statistical analyses of in vitro studies were performed with the “XLSTAT add-in” software. After a Shapiro–Wilk normality test, data not showing a Gaussian distribution were analyzed with the Kruskal–Wallis test, the post ad hoc Dunn’s multiple comparison test and Bonferroni correction (>2 experimental groups). Statistical tests applied to in silico investigations are described in the respective sections.

## 3. Results

### 3.1. Refinement of the AMD-Associated rs704 Signal at the VTN Gene Locus by Subgroup Analysis

**Figure 1:**
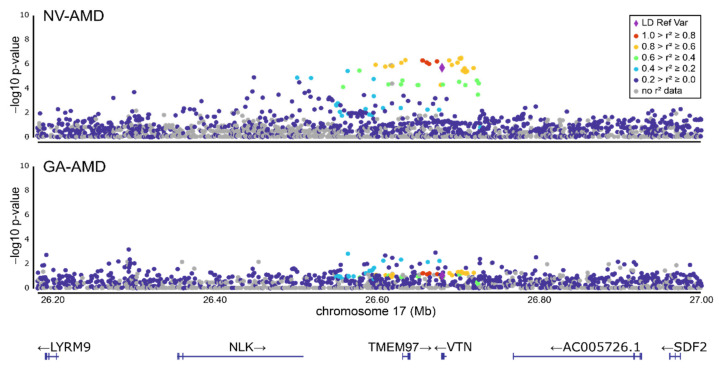
Association of genetic variant rs704 with late-stage AMD subtypes neovascular AMD (NV-AMD) and geographic atrophy (GA-AMD). The association of rs704 with AMD was calculated separately for both late-stage disease phenotypes in the IAMDGC dataset. While an association signal with AMD was detected in the NV subgroup, an association in GA samples is not evident. Variant rs704 is presented as purple diamond and serves as linkage disequilibrium (LD) reference variant. Transcript map with direction of transcription is given at the bottom. Plots were generated with Locuszoom [26,27].

To further delineate the AMD association signal at 17q11.2 (*TMEM97*/*VTN* locus), we performed an AMD subgroup analysis, separating early and late stages of the disease. This showed an association of rs704 with late-stage AMD (Q-value = 1.79 × 10^−5^), confirming an earlier report [4]. No association was seen with early AMD (Table 1). Furthermore, the association with late-stage AMD was refined by specifying subgroups of individuals presenting with either GA-, NV- or a combined phenotype of GA- and NV-AMD. This revealed a significant association of rs704 with NV-AMD (Q-value = 1.79 × 10^−5^), but not with GA-AMD (Q-value = 0.118) or the combined phenotype of GA- and NV-AMD (Q-value = 0.135). It should be noted that the number of individuals in the GA and the combined GA and NV subgroup are somewhat smaller than in the NV cohort. This may lead to a slightly reduced statistical power in this dataset (Table 1, Figure 1). When the association was calculated separately for males and females, a comparable nominal association of rs704 was found in both genders (males: Q-value = 2.17 × 10^−^^3^; females: Q-value = 2.17 × 10^−3^).

### 3.2. Functional Relationship between Vitronectin and Angiogenesis Regulator PAI-1

The association of rs704 exclusively with the NV-AMD phenotype could reflect a pathogenic consequence of this variant on vitronectin function that may be closely linked to pathophysiological mechanisms distinctive to this disease subtype, in particular neovascularization [2,39]. To explore the contribution of rs704 and vitronectin in NV-AMD, we specifically focused on the functional relationship between vitronectin and its isoforms with the known interaction partner PAI-1 [40]. PAI-1 is a crucial regulator in the progression of vascular events [41], and as such was already implicated in NV-AMD [42,43,44]. Initially, the association of genetic variation in or near the *PAI-1* gene at 7q21.3–q22 was assessed in the IAMDGC dataset and revealed a signal with nominal significance at this locus, exclusively carried by the NV-AMD association (variant with smallest *p*-value rs28709821, *p* = 2.71 × 10^−6^) (Supplementary Figure S3).

### 3.3. Genetic Variant rs704 and Vitronectin Binding to PAI-1

**Figure 2:**
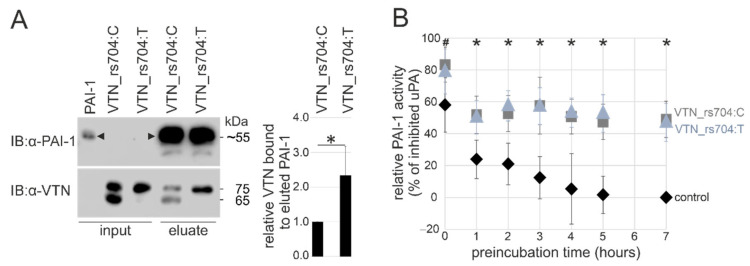
Effect of rs704 on vitronectin capacity to bind PAI-1 and maintain its active state. (**A**) Affinity chromatography columns coupled with purified recombinant PAI-1 (input) were loaded with recombinant VTN_rs704: C (input) or VTN_rs704: T (input). After several washing steps, proteins were eluted and subjected to immunoblot (IB) analysis with antibodies against vitronectin (α-VTN) and PAI-1 (α-PAI-1). Black arrowheads indicate the recombinant PAI-1 at a major molecular weight species slightly above 55 kDa, which results from the addition of Strep-tag (5.1 kDa) to the glycosylated PAI-1 (50–54 kDa, [46,47]). Smaller molecular weight species are also visible, as previously reported for purified PAI-1 [30,31]. After densitometric quantification, vitronectin signals were normalized for the respective inputs and eluted PAI-1. Data represent the mean ± standard deviation (SD) of five replicates, calibrated against VTN_rs704: C. Asterisks indicate statistically significant differences (* *p* < 0.05, Mann–Whitney U test). (**B**) VTN_rs704: C (grey squares), VTN_rs704: T (ice-blue triangles), or control eluate (control, black diamonds) were mixed with PAI-1 and incubated at 37 °C. Aliquots of vitronectin/PAI-1 or control eluate/PAI-1 mixtures were subjected to the PAI-1 activity assay after 0, 1, 2, 3, 4, 5 and 7 h, measuring PAI-1-dependent uPA activity. The data were calibrated against the values obtained for control measurements at 7 h (uPA at its maximum activity in our experimental setting, 100%). To better visualize the extent of PAI-1 inhibition and thus the inhibitory capacity of PAI-1, the calibrated uPA activity was subtracted from 100% (maximal uPA activity). Data represent the mean ± SD of six replicates, calibrated against control at 7 h. Asterisks indicate statistically significant differences (* *p* < 0.05, Kruskal–Wallis test, followed by Dunn’s multiple comparison test with Bonferroni correction) between control and VTN_rs704: C as well as between control and VTN_rs704: T. Hash character indicates statistically significant difference (^#^ *p* < 0.05, Kruskal–Wallis test, followed by Dunn’s multiple comparison test with Bonferroni correction) between VTN_rs704: C and control.

In co-precipitation experiments, non-AMD risk-associated VTN_rs704: C and AMD risk-associated VTN_rs704: T were incubated with immobilized recombinant PAI-1. After several washing steps and elution of PAI-1, Western blot analysis demonstrated co-elution of both vitronectin isoforms with PAI-1 (Figure 2A; Supplementary Figure S2A). A control experiment without immobilized PAI-1 failed to detect vitronectin signals in the elution fraction, while instead, vitronectin was predominantly found in the flow-through and washing fractions (Supplementary Figure S2C). Interestingly, densitometric quantification revealed higher levels of VTN_rs704: T in the elution fraction compared to VTN_rs704: C (2.28 ± 0.81-fold increase, *p* < 0.05; Figure 2A).

### 3.4. Genetic Variant rs704 and the Capacity of Vitronectin to Stabilize PAI-1 Activity

PAI-1 spontaneously converts from the enzymatically active state to an energetically more favorable but non-functional conformation [36]. Under physiological conditions, this transition occurs with a half-life of approximately two hours at 37 °C. Through allosteric modulation, vitronectin stabilizes active PAI-1 by slowing its latency conversion [36]. The capacity of VTN_rs704: C and VTN_rs704: T to preserve the active PAI-1 form was addressed in a cell-free enzymatic assay by assessing the ability of PAI-1 to inhibit the proteolytic activity of its target protease uPA [36,45], and, thus, the uPA-mediated cleavage of its chromogenic substrate. PAI-1 was preincubated with or without VTN_rs704: C or VTN_rs704: T for 0, 1, 2, 3, 4, 5, and 7 h to test the stabilization of active PAI-1 by the vitronectin isoforms. After preincubation, the PAI-1-containing samples were first mixed with uPA for 30 min, and then subjected to the PAI-1 activity assay, measuring the cleavage of a chromogenic substrate by uPA after 2 h of incubation. As depicted in Figure 2B, both vitronectin isoforms stabilized the enzymatically active PAI-1, and thus increased the PAI-1-induced inhibition of uPA, with no statistically significant difference between VTN_rs704: C and VTN_rs704: T. In contrast, PAI-1 incubated without vitronectin showed a strong and time-dependent decrease in its ability to inhibit uPA (Figure 2B). Both vitronectin isoforms showed an immediate effect in stabilizing PAI-1 and thus enhancing the inhibitory activity of PAI-1 (uPA inhibition by PAI-1 at 0 h: 83 ± 11% in the presence of VTN_rs704: C, 79 ± 14% in the presence of VTN_rs704: T, 58 ± 17% in the presence of control eluate, Bonferroni-adjusted *p* < 0.05 between VTN_rs704: C and control, Figure 2B). The stabilizing effect of vitronectin on PAI-1 was still effective after 7 h of preincubation, while in samples without vitronectin, PAI-1 strongly lost its inhibitory function (uPA inhibition by PAI-1 at 7 h: 49 ± 11% in the presence of VTN_rs704: C and 47 ± 12% in the presence of VTN_rs704: T compared to 0% in the presence of control eluate; Bonferroni-adjusted *p* < 0.05 between control and VTN_rs704: C or VTN_rs704: T; Figure 2B). An independent series of analyses testing 72 h of preincubation revealed almost identical results to the 7 h preincubation: no PAI-1 activity in the samples without vitronectin and comparable results for both isoforms of vitronectin in stabilizing PAI-1 with a similar effect size from 1 to 7 h of preincubation (Supplementary Figure S4). The observed stabilization of PAI-1 by vitronectin at 0 h of preincubation is likely due to a stabilizing effect of vitronectin during the further 2.5 h of incubation required for the enzymatic reaction.

### 3.5. Vitronectin and Cellular PAI-1 Protein Expression and ECM Deposition

**Figure 3:**
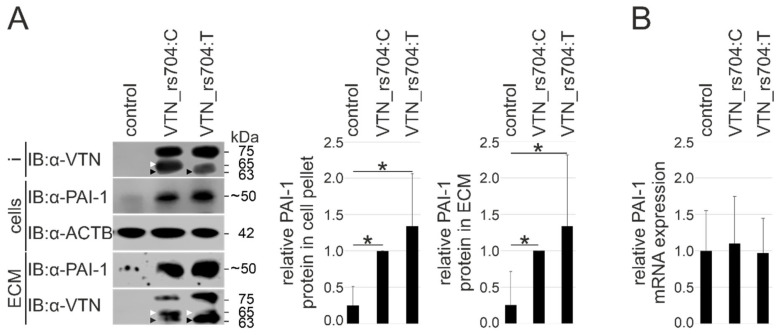
Effect of vitronectin isoforms on endogenous endothelial PAI-1 protein expression and deposition. HUVECs were incubated for 24 h with input (i) including VTN_rs704: C, VTN_rs704: T, or the same volume of control eluate (control), respectively. (**A**) Input, cells and ECMs were subjected to immunoblot (IB) analysis with antibodies against vitronectin, PAI-1 and ACTB. Endogenous PAI-1 is recognizable as a major molecular weight species of approximately 50 kDa, consistent with previous reports of glycosylated PAI-1 as a 50–54 kDa molecular weight species [46,47]. Arrowheads indicate different fractions of recombinant vitronectin after its proteolytic cleavages (white arrows: cleavage between aa Arg398 and Ala399, black arrows: cleavage between aa Arg380 and Ser381) by endogenously expressed endoproteases. In contrast to VTN_rs704: C that stains as a mixture of a single polypeptide chain (about 75 kDa) and a clipped form (two chains of 65 and 10 kDa, held together by disulfide bonds, with the 10 kDa chain not detectable within this experimental setup), VTN_rs704: T is less susceptible to the proteolytic cleavage at Arg398-Ala399 indicated by a higher proportion of the uncleaved 75 kDa protein [8,32]. Molecular weights of approximately 63 kDa (black arrows) depict vitronectin fragments resulting from cleavage at Arg380-Ser381, as described in [50]. PAI-1 signals were densitometrically quantified and normalized against ACTB. Data represent the mean ± SD of nine biological replicates, calibrated against the PAI-1 signals obtained after VTN_rs704: C addition. Asterisks indicate statistically significant differences (* *p* < 0.05, Kruskal–Wallis test, followed by Dunn’s multiple comparison test with Bonferroni correction). (**B**) mRNA was isolated and *PAI-1* expression determined by qRT-PCR. Data represent the mean ± SD of six biological replicates, calibrated against the control.

Vascular endothelial cells are among the most common source of PAI-1 expression [48,49]. In this study, the effect of vitronectin isoforms on endogenous PAI-1 and ECM deposition was investigated in endothelium-derived HUVECs. Cells were incubated with VTN_rs704: C, VTN_rs704: T, or control eluate for 24 h. Subsequent Western blot analysis revealed an approximately 4-fold higher concentration of endogenous PAI-1 in cellular lysates exposed to the vitronectin isoforms compared to the control eluate (Bonferroni-adjusted *p* < 0.05 between control and both vitronectin isoforms, VTN_rs704: C and VTN_rs704: T; Figure 3A). Concomitant with a precipitation of both vitronectin isoforms initially added to the supernatant in the ECM deposited by HUVECs (Figure 3A), an approximately 4-fold higher accumulation of extracellular PAI-1 was observed in the ECM of vitronectin-treated cells compared to control-treated cells (Bonferroni-adjusted *p* < 0.05 between control and both vitronectin isoforms, VTN_rs704: C and VTN_rs704: T; Figure 3A). To investigate whether the increase in PAI-1 protein expression in vitronectin-treated cells could be a consequence of enhanced PAI-1 gene expression, PAI-1 transcription was quantified in HUVECs treated with vitronectin isoforms or control eluate by qRT-PCR. After 24 h, no difference was evidenced in PAI-1 expression between cells treated with VTN_rs704: C, VTN_rs704: T, or control eluate (Figure 3B).

**Figure 4:**
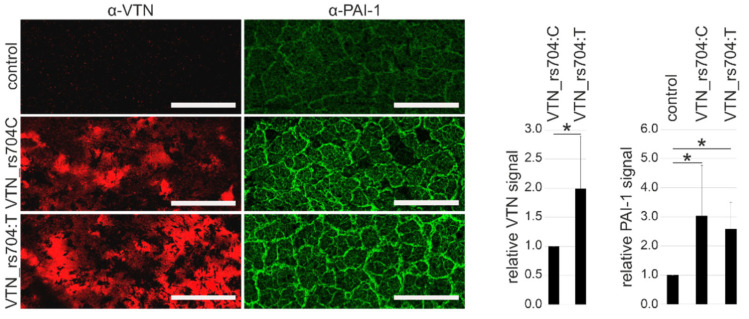
Effect of vitronectin isoforms on ECM deposition of PAI-1 by RPE-derived cells. ARPE-19 cells transfected with expression vectors for VTN_rs704: C or VTN_rs704: T or an empty control vector (pcDNA3.1, control) were seeded onto transwell filter inserts. After 8 weeks, the inserts were decellularized and subjected to immunofluorescence staining with antibodies against vitronectin (α-VTN) and PAI-1 (α-PAI-1). Confocal microscopy images were taken at 10× magnification. Scale bars: 100 µm. Data represent the mean ± SD of four independent replicates, calibrated against VTN_rs704: C (for vitronectin signals) or control (for PAI-1 signals). Asterisks indicate statistically significant differences (* *p* < 0.05, Mann–Whitney U test for VTN; * *p* < 0.05, Kruskal–Wallis test for PAI-1, followed by Dunn’s multiple comparison test with Bonferroni correction).

Furthermore, the effect of VTN_rs704: C and VTN_rs704: T on PAI-1 deposition to the ECM was also explored in an alternative cell type: the RPE-derived cell line ARPE-19 [51]. Cells were transfected with expression vectors for the two untagged vitronectin isoforms, or with an empty control vector, and incubated on transwell filters for 8 weeks. After immunolabeling, decellularized ECM produced by ARPE-19 cells heterologously expressing vitronectin showed an approximately 3-fold increase in PAI-1 protein expression (3.03 ± 1.73 with VTN_rs704: C, 2.59 ± 0.91 with VTN_rs704: T, compared to control; Bonferroni-adjusted *p* < 0.05 between control and VTN_rs704: C or VTN_rs704: T; Figure 4). The ECMs revealed an increased accumulation of vitronectin (Figure 4), which was significantly more pronounced for the VTN_rs704: T isoform than for VTN_rs704: C (1.99 ± 0.90-fold increase; *p* < 0.05), in line with our previous findings in ECM depositions of transfected ARPE-19 cells after 4 weeks of cultivation [8].

### 3.6. In Silico VTN and PAI-1 Expression Profiles

**Figure 5:**
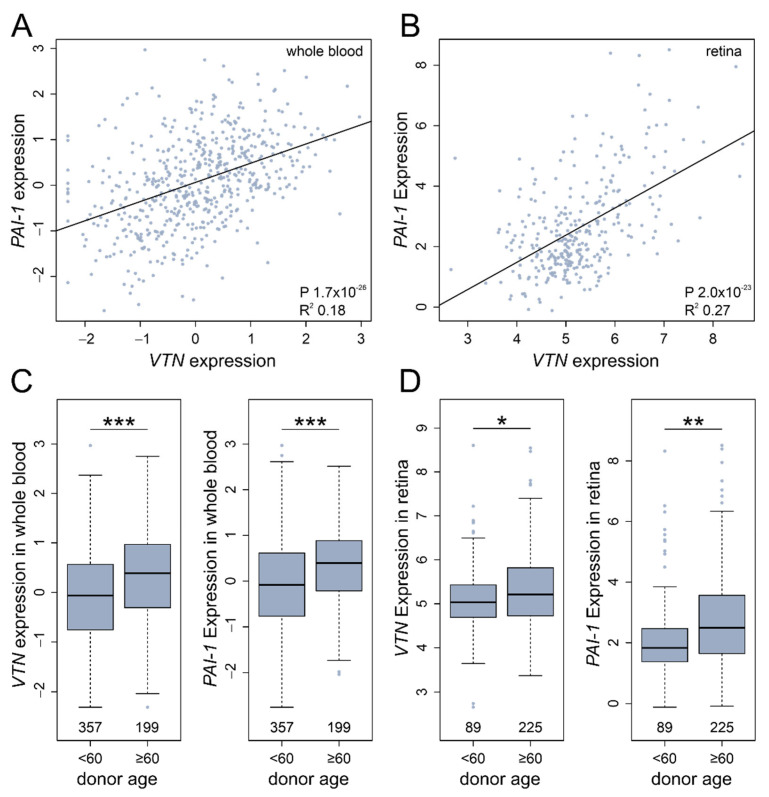
*VTN* and *PAI-1* expression in whole blood and retinal tissue. Linear regression models were applied to analyze the relationship of *VTN* and *PAI-1* mRNA gene expression in (**A**) whole blood and (**B**) retinal tissue. The 556 whole blood samples were derived from European donors in the GTEx dataset and underwent several normalization steps, as described in [20,21]. The analysis in retina was based on 311 healthy retina samples, as described in [22]. (**C**) Gene expression of 357 whole blood samples retrieved from donors below the age of 60 and 199 samples from individuals at or over 60 years of age. *VTN* and *PAI-1* were significantly upregulated in the older cohort. (**D**) Similar effects, although less pronounced, were obtained in gene expression analyses of retinal tissues. (Mann–Whitney U test, * *p*< 0.05, **p < 0.01, *** *p* < 0.001).

An analysis of the functional relationship between vitronectin and PAI-1 was extended to delineate the mRNA profiles in silico by using a large-scale correlation approach. The analysis of *VTN* and *PAI-1* mRNA expression from whole blood samples (*n* = 556) [20] and healthy retinal tissue (*n* = 311) [22] revealed a positive correlation between *VTN* and *PAI-1* expression (whole blood, adj. R^2^: 0.18, *p* = 1.73 × 10^−26^; retina, adj. R^2^: 0.27, *p* = 1.99 × 10^−23^; Figure 5A,B). A linear regression model of donor age and gene expression demonstrated that *VTN* and *PAI-1* transcription is significantly increased in samples retrieved from older donors in both tissues analyzed (linear model: *VTN*, whole blood *p* = 5.05 × 10^−6^; retina *p* = 0.047; *PAI-1*, whole blood *p* = 1.11 × 10^−5^; retina *p* = 0.025; Supplementary Figure S5). This effect is even stronger when comparing individuals in the categories “above 60” and “below 60” years of age. Individuals older than 60 years showed a higher expression of *VTN* and *PAI-1* in whole blood in comparison to younger individuals (*VTN*, *p* = 4.01 × 10^−6^; PAI-1, *p* = 1.64 × 10^−6^; Mann–Whitney U test; Figure 5C). A similar result, although less pronounced, was obtained for the retinal tissues: a significant increase in *VTN* and *PAI-1* expression was detectable in individuals older than 60 years, when compared to younger individuals (*VTN*, *p* = 0.039; *PAI-1*, retina *p* = 0.0009; Mann–Whitney U test; Figure 5D).

## 4. Discussion

Our aim was to consolidate the association of the non-synonymous variant rs704 in the *VTN* gene with AMD and its phenotypic subtypes and to gain deeper insight into the involvement of rs704 vitronectin isoforms in AMD pathogenesis. Consequently, we can confirm the previously reported genetic association of rs704 with late-stage AMD [4] and demonstrate that this association is almost exclusively carried by the NV-AMD subtype. Consistent with findings in an earlier study by our group, where we demonstrated the significant effect of rs704 on vitronectin protein expression and processing, and a role in angiogenesis-related processes such as ECM remodeling and cell migration [8], we now focused on the rs704-related vitronectin isoforms (non-AMD risk-associated VTN_rs704: C and AMD risk-associated VTN_rs704: T) and their interaction with the angiogenic regulator PAI-1. The ability of vitronectin to bind PAI-1 was influenced by rs704, while the capacity to stabilize the active form of PAI-1 was not. In cultured endothelial cells and RPE cell line ARPE-19, the presence of both vitronectin isoforms induced a strong accumulation of PAI-1 in the ECM. Interestingly, a large-scale gene expression analysis revealed an age-dependent simultaneous increase in both *VTN* and *PAI-1* mRNA expression. Together, our data outline a novel biological mechanism in which age- and genotype-dependent vitronectin and PAI-1 dysregulation may contribute to the development of NV-AMD.

Late-stage subtypes NV-AMD and GA-AMD mainly share a common genetic predisposition [4]; only few variants have distinctly been associated with only one form of late-stage disease [4,52]. This has promoted the idea that the late stages of disease largely underly common pathways. The first NV-specific variant was found near the matrix metalloproteinase gene *MMP9*, which is known to be implicated in ECM degradation processes [4]. The current study now adds a further NV-specific variant, namely rs704, pointing to an involvement of vitronectin and vitronectin-related pathways in AMD-associated vascular processes [53].

Vitronectin action in the regulation of endothelial cell adhesion/invasion and ECM remodeling is mediated by directly interacting with cell surface receptors involved in intracellular signaling, or with a number of specific extracellular proteins and growth factors [9,10,11,14,15,54,55,56]. Among the various known interaction partners of vitronectin is PAI-1, which appears to be a particularly attractive candidate in our study, as PAI-1 is known to be a pivotal factor in vascular events associated with degenerative diseases of the retina such as AMD [42,43,44,57,58]. Moreover, transgenic mice overexpressing human PAI-1 in retinal microvasculature revealed thickening of the endothelial ECM surrounding the retinal vessels and altered the endothelial cell-pericyte ratio [59], processes also connected to vascular changes in AMD [60,61,62]. The activity of PAI-1, and thus its regulation of angiogenic processes such as pericellular proteolysis, cell adhesion and reattachment, as well as the initiation of related intracellular signaling pathways [63,64,65], is strictly dependent on vitronectin binding [36].

Our analysis of the non-synonymous variant rs704 revealed a stronger binding of the AMD risk-associated isoform of vitronectin (VTN_rs704: T) to PAI-1, compared with the non-risk-associated isoform (VTN_rs704: C). This seems to contradict the results by Gibson and colleagues, which suggest that rs704 has no impact on the binding of vitronectin to PAI-1 [66]. Their study, however, used a truncated version of VTN_rs704: C, apparently lacking the short chain peptide of the rs704-dependent protein cleavage product, although the short fragment in VTN_rs704: C remains bound to the protein by a disulphide bond in vivo [8,67,68] and likely influences the folding or accessibility of putative binding sites for PAI-1. The stronger binding of VTN_rs704: T to PAI-1 may be explained by the rs704-dependent amino acid change (Thr400Met), which causes altered protein folding and processing [32,69], potentially influencing accessibility to the PAI-1 interaction site. Alternatively, the altered binding properties could reveal specificities in the PAI-1-mediated assembly into higher-order complexes [70,71]. This could affect the number of vitronectin monomers complexed, and thus retained, by immobilized PAI-1.

Of note, the two rs704 vitronectin isoforms revealed no difference in the capacity to stabilize active PAI-1. We speculate that the co-precipitation assay measures stable physical interaction between vitronectin and PAI-1, whereas the enzymatic assay may also detect less stable, unstable, or possibly even transient interactions. A stable binding between vitronectin and PAI-1 might not be relevant for stabilizing active PAI-1, as stable interactions between two biomolecules is not necessarily a requirement for enzymatic processes. For example, rhomboid proteases cleave their substrate highly efficiently without exhibiting an apparent binding affinity [72]. Alternatively, different sensitivities of the two experimental approaches or a saturation effect in the enzymatic assay may account for the apparently conflicting results.

Interestingly, our experiments show that HUVECs significantly increase PAI-1 protein expression in cellular fractions and ECM upon externally added recombinant vitronectin isoforms. There was also a tendency towards an increased PAI-1 protein level in cells and ECM after exposure to VTN:rs704: T compared to VTN_rs704: C, although this was not statistically significant. Likewise, these effects were also seen in ARPE-19 cells. Consistent with our observations, studies reported an increase in endothelial PAI-1 deposition by human umbilical arterial endothelial cells (HUAECs) and HUVECs grown on vitronectin, compared to other ECM substrates [73,74].

Eukaryotic protein expression and processing are dependent on numerous cellular sequences of events such as, e.g., transcription, mRNA decay, protein modification, processing and degradation, localization, as well as signal transduction [75,76]. Since *PAI-1* transcription in HUVECs remained unchanged after exposure to vitronectin, the increased amount of PAI-1 protein may be attributed to a number of posttranslational processes such as altered protein localization, altered intracellular processing or export, changes in stability, internalization, or signal transduction.

In cultured blood monocytes, a mechanism by which vitronectin prevents PAI-1 degradation has been described [77]. When bound to vitronectin, active PAI-1 undergoes a conformational change that makes it less accessible to proteolytic enzymes. The observed increase in ECM-bound PAI-1 in the presence of vitronectin might also be explained by the capacity of vitronectin to translocate PAI-1 into the ECM, as it was shown that vitronectin mediates the binding of PAI-1 to the ECM [78]. Furthermore, it was reported that active PAI-1 (which increases in the presence of vitronectin, as discussed above) is internalized when complexed with uPA via receptor-mediated endocytosis [77,79]. This could explain the increase in cellular PAI-1 after the incubation of HUVECs with vitronectin. Finally, increased extracellular amounts of PAI-1 (obtained after proteolytic protection by vitronectin, as suggested by [77]) could also lead to increased binding of PAI-1 to the cell surface of HUVECs, e.g., via the low-density lipoprotein receptor-related protein (LRP) [77,80], which could account for the increased amount of PAI-1 in HUVEC cell fractions as found in our study. To our knowledge, there is no report on intracellular post-translational modifications of PAI-1 (e.g., via glycosylation or phosphorylation) induced by vitronectin.

Notably, in vitro analyses only permit limited conclusions on the actual relevance to molecular pathomechanisms of complex diseases such as AMD. The cultivation of cells in plastic without tissue context, physiological blood supply, physiological concentrations of oxygen, carbon dioxide and other gases, times of disease duration of mostly days to weeks instead of decades, no endogenous or exogenous stressors such as ageing, oxidative stress, inflammation, etc., render cell culture systems, to some extent, rather artificial. Nevertheless, in vitro cellular models allow us to directly evaluate the effects of individual components without the putative masking effect or interference of similar components, in this case, e.g., other matricellular or angiogenesis-related proteins. The HUVEC cell line is well-established for studying pathogenic processes related to vascular malfunction, also in AMD [81,82,83], although there may be cellular model systems more closely fitting to choroid-retinal endothelial cells, such as the endothelial macaque-derived cell line RF/6A [84]. In our study, however, we aimed to investigate the interplay between vitronectin and PAI-1 on a more systemic/broader level, given that in addition to local production [5,16,85,86], major proportions of PAI-1 and vitronectin in the retina are likely derived from the blood circulation. Of note, systemic bloodstream-derived PAI-1 has been shown to affect the caliber of retinal vessels [87]. Additionally, a major proportion of vitronectin in Bruch’s membrane and in drusen originate from the systemic bloodstream and, thus, from extraretinal/extrachoroidal origin [88,89]. Increased levels of plasma-derived vitronectin have been detected in eyes with retinal pathologies such as proliferative diabetic retinopathy [90]. Finally, HUVECs are a well-established and valuable model-system to specifically study functional aspects of PAI-1 due to their endogenous high PAI-1 expression [91,92,93].

ARPE-19 cells were chosen for the present study, as these cells are known to produce an ECM consisting of typical Bruch’s membrane proteins [37,94]. Furthermore, ARPE19-derived ECM reveals distinctive functional aspects of Bruch’s membrane, showing a stimulating effect on RPE differentiation of primary RPE cells, as well as on viability, attachment, and mitochondrial activity of hiPSC-derived RPE cells [37,95]. While ARPE-19 cells may exhibit only a roughly hexagonal shape, as well as limited pigmentation, transepithelial resistance and polarisation [96,97], these cells are still a valuable and frequently used model for analyzing Bruch’s membrane pathology. While other RPE cell models such as hiPSC-RPE cells show a more pronounced RPE-like phenotype, they are rather inconvenient for sufficient transfection efficiency required for the long-term study on the effects of the different vitronectin isoforms on PAI-1 deposition into the ECM. Furthermore, long-term cultivation of hiPSC-RPE cells on filters requires serum replacement and coating with a basement membrane matrix, a proteinaceous surface containing numerous ECM proteins. These supplements contain a non-described mixture of proteins, nutrients, and other components, which may mask the effects of vitronectin. In contrast, ARPE-19 cells are simple to cultivate on uncoated filters in medium without serum or serum replacement, allowing a rather “background-free” investigation of the effect of vitronectin overexpression on PAI-1 deposition.

Finally, our large-scale gene expression analysis addressed the age-dependency of AMD symptoms and uncovered an increased mRNA transcription of both *VTN* and *PAI-1* in human retinal and blood tissues with increasing age, documenting a statistically significant difference in samples aged 60 years and older. It should be noted that the linear statistical regression model used in our study to analyze in silico expression is limited merely to measuring the strength and direction of the relationship between two variables (i.e., *VTN* and *PAI-1* mRNA expression) and calculating their association. Consequently, such analyses do not provide an explicit causal interpretation [98]. Results of our gene expression analysis of HUVECs subjected to vitronectin suggest no direct impact of vitronectin on *PAI-1* gene expression. However, the expression of a wide range of genes is altered during ageing, either through the influence or induction of a variety of cellular processes, e.g., mitochondrial dysfunction, low-grade inflammation, senescence or defective proteostasis [99,100,101]. The observed age-dependent transcriptional changes in *VTN* and *PAI-1* could thus be independently induced by age-related processes. For example, vitronectin functions as an acute-phase molecule, exhibiting increased tissue levels under inflammatory conditions [102,103,104], which also increase with age [101]. Likewise, PAI-1 is dramatically elevated in the proinflammatory state, as in tissue injury, sepsis, and inflammation [105], and is also considered a key marker and mediator of senescence [106], a hallmark of ageing [101]. Thus, *VTN* and *PAI-1* gene expression may be upregulated cooperatively by age-related mechanisms, but without a mutual dependency of each other. Given that age is a major risk factor for the development of AMD [1,107], the increased expression of these two regulators of angiogenesis with age would be consistent with a role for vitronectin and PAI-1 in the development of AMD.

**Figure 6:**
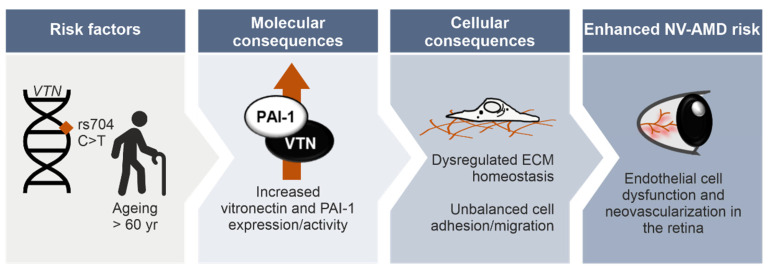
Conceptual disease model of rs704-associated pathobiology leading to NV-AMD.

Our findings, in conjunction with previous observations [8,42,44,108], led us to propose a conceptual disease model for neovascular AMD (Figure 6), in which the risk-associated rs704: T genotype in combination with ageing would lead to a significant increase in vitronectin and PAI-1 protein expression, eventually contributing to vascular changes in this late-stage AMD phenotype. Specifically, the risk-associated rs704: T genotype is expected to increase endogenously expressed vitronectin, as shown in our previous study [8] and reported by Sun and colleagues in their proteomic analyses of human plasma [108]. Given the close correlation discussed above between the amount/activity of PAI-1 and the presence of vitronectin, increased vitronectin protein expression should raise the concentration and activity of PAI-1. Interestingly, VTN_rs704: T also showed a somewhat higher binding affinity for PAI-1. The rs704-induced alteration in protein expression and activity of both vitronectin and PAI-1 could be aggravated by a gradual increase in the gene expression of *VTN* and *PAI-1* with age.

In the retina, vitronectin could concentrate local PAI-1 and its activity in the vicinity of endothelial cells. This would depend on the rs704 genotype and age. In line with this, Basu and colleagues observed in oxygen-induced murine retinopathy that the role of PAI-1 in retinal angiogenesis is enhanced by the presence of vitronectin in regions where new vessels form [58]. In addition to coordinating local PAI-1, vitronectin could potentially be a vehicle for PAI-1 from the bloodstream, given that PAI-1 in blood mainly circulates in a complex with plasma vitronectin [40,109]. Due to vitronectin transcytosis by vascular endothelial cells [110], vitronectin could direct complexed PAI-1 from the bloodstream into the subendothelial matrix, as well as into the extracellular space adjacent to the RPE. Subsequently, altered retinal concentrations of both proteins may ultimately affect the subtle balance between the angiogenesis-associated processes regulated by these two proteins.

## 5. Conclusions

In conclusion, our findings from in silico analyses and in vitro experiments shed new light on the involvement of vitronectin to the pathogenesis of AMD and suggest a vitronectin- and PAI-1-dependent biological mechanism, possibly promoting the neovascular complications seen in NV-AMD. The delineation of this pathway could facilitate the development of diagnostic, preventive, or therapeutic treatment strategies. Vitronectin has been recognized previously as a promising therapeutic target to regulate complement activation and inflammation [111] and to prevent ectopic deposits associated with AMD [19]. Addressing the need to regulate and not simply inhibit pathological angiogenic events in NV-AMD [112], therapeutic modulation of vitronectin and PAI-1 protein expression, either through genetic or pharmacological approaches, could contribute to the restoration of vascular homeostasis and, thus, help to prevent or treat vascular abnormalities in late stage AMD.

## Figures and Tables

**Table 1:** Association of rs704 with AMD subtypes.

	Cases (*n*)	Controls (*n*)	OR [95% CI]	*p*-Value	Q-Value
Early stage AMD	6.657	17.832	0.978 [0.939–1.018]	0.286	0.286
Late-stage AMD	16.144	17.832	1.078 [1.044–1.113]	3.97 × 10^−6^	**1.79 × 10^−5^**
Geographic atrophy (GA)	3.235	17.832	1.048 [0.992–1.106]	0.092	0.118
Neovascularization (NV)	10.749	17.832	1.09 [1.052–1.13]	2.02 × 10^−6^	**1.79 × 10^−5^**
Combined, GA & NV	2.160	17.832	1.053 [0.987–1.124]	0.120	0.135
Male	6.532	7.820	1.084 [1.034–1.139]	1.03 × 10^−3^	2.17 × 10^−3^
Female	9.612	10.012	1.073 [1.027–1.119]	1.21 × 10^−3^	2.17 × 10^−3^

Association of rs704 with AMD subtypes in the IAMDGC dataset. For all associations, the T allele indicates the effect allele. Associations were calculated in comparison with non-AMD individuals (controls). Q-values < 0.05 are indicated in bold.

## Data Availability

The data permitted for sharing by the respective institutional review boards from the IAMDGC are available at the database of genotypes and phenotypes (dbGAP) under the accession number phs001039.v1.p1. The gene expression data used for the analyses described in this manuscript were obtained from the GTEx Portal and via dbGAP accession number phs000424.v8.p2. Processed gene expression data of GTEx can be downloaded at the GTEx Portal [113].

## Supplementary Materials

The following supporting information can be downloaded at: https://www.mdpi.com/article/10.3390/cells11111766/s1. Table S1: Primer sequences, targets and applications in expression cloning or real-time quantitative RT-PCR; Table S2: Antibodies, dilutions, applications, and origins; Figure S1: Purification of Strep-tagged PAI-1, VTN_rs704:C and VTN_rs704:T; Figure S2: Controls for co-precipitation experiments; Figure S3: Association of the genetic locus harboring the *PAI-1* gene with late-stage AMD phenotypes and the subgroups of neovascular AMD (NV-AMD) and geographic atrophy (GA-AMD); Figure S4: PAI-1 activity assay after 0 and 72 h preincubation of PAI-1 and vitronectin; Figure S5: Gene expression of *VTN* and *PAI-1* in human blood and retina separated by age groups.
