# Supplementary material for: Vitronectin and Its Interaction with PAI-1 Suggests a Functional Link to Vascular Changes in AMD Pathobiology
Source: Cells. 2022 May 27;11(11):1766. doi: 10.3390/cells11111766 (PMC9179922; doi:10.3390/cells11111766)
Supplement: Supplementary file 1 [file cells-11-01766-s001.zip › cells-1711685-supplementary.pdf]

Supplementary Material

Vitronectin and its interaction with PAI-1 suggests a functional link to vascular changes in AMD pathobiology

Fabiola Biasella [1], Tobias Strunz [1], Christina Kiel [1], International AMD Genomics Consortium (IAMDGC) [2], Bernhard H. F. Weber [1, 3] and Ulrike Friedrich [1]

[1] Institute of Human Genetics, University of Regensburg, Franz-Josef-Strauss-Allee 11, 93053 Regensburg, Germany

[2] John P. Hussman Institute for Human Genomics, University of Miami, Miami, FL 33136, USA

[3] Institute of Clinical Human Genetics, University Hospital Regensburg, Franz-Josef-Strauss-Allee 11, 93053 Regensburg, Germany

Correspondence: U.F. Ulrike.Friedrich@klinik.uni-regensburg.de; Tel.: +49 941-944-5428; Fax: +49 941-944-5402; B.H.F.W. bweb@klinik.uni-regensburg.de; Tel.: +49 941-944-5400; Fax: +49 941-944-5402

Supplementary Table S1. Primer sequences, target and application in expression cloning or real-time quantitative PCR.

**Target**	**Primer/probe name**	**Primer/probe Sequence (5'-3')**	**Application**	**Polymerase/ MasterMix**
PAI-1	PAI-1-EcoRI-F	**F:**gaattcATGCAGATGTCTCCAGCCCTCA	Cloning of pEXPR-IBA103-PAI-1	GoTaq® DNA Polymerase (Promega Corporation, Madison, WI, USA)
	PAI-1-XhoIwostop-R	**R:**ctcgagGGGTTCCATCACTTGGCCCAT		
	PAI-1-seq1-F	**F:**acagacgcgatcttggtcca	Sequencing of pEXPR-IBA103-PAI-1	AmpliTaq® DNA Polymerase (BigDye Terminator v1.1 Cycle Sequencing Kit, Thermo Fisher)
	PAI-1-seq2-F	**F:**TATACTGAGTTCACCACGCCCG		
Expression vector (flanking cloning sites)	pEXPR-IBA3-F	**F:**GAGAACCCACTGCTTACTGGC		
	pEXPR-IBA3-R	**R:**TAGAAGGCACAGTCGAGG		
PAI-1	FH1_SERPINE1	**F:**GCTGCAGAAAGTGAAGATCG	Real-Time quantitative PCR	Takyon™ DNA Polymerase (Takyon™ Low ROX Probe 2X MasterMix dTTP blue; Eurogentec, Seraing, Liège, Belgium)
	RH1_SERPINE1	**R:**GTCCATGATGATCTCCTCGG		
	PH1_SERPINE1	**P:**(6FAM)GTGGCCTCCTCATCCAACAGCTGTC(OQA)		
HPRT1	FH1_HPRT1	**F:**CTTTGCTTTCCTTGGTCAGG		
	RH1_HPRT1	**R:**TCAAATCCAACAAAGTCTGGC		
	PH1_HPRT1	**P:**(6FAM)GCTTGCTGGTGAAAAGGACCCCACG(OQA)		

F: Forward; R: Reverse; P: Probe

Supplementary Table S2. Antibodies, their dilution, application, and origin.

**Antibodies**	**Dilution**	**Application**	**Manufacturer (Country)**
α-VTN<br>(MAB2349)	1:5000<br>1:250	WB<br>IF	R&D Systems, Inc., Minneapolis, MN, USA
α-PAI-1<br>(11907S)	1:1000<br>1:100	WB<br>IF	Cell Signaling Technology, Danvers, MA, USA
α-ACTB<br>(A5441)	1:10000	WB	Sigma-Aldrich, St. Louis, MO, USA
Goat Anti-Mouse IgG,<br>Peroxidase conjugated	1:10000	WB	(Calbiochem) Merck Chemicals GmbH, Darmstadt, Germany
Goat Anti-Rabbit IgG,<br>Peroxidase conjugated	1:10000	WB	(Calbiochem) Merck Chemicals GmbH, Darmstadt, Germany
Goat Anti-Mouse IgG,<br>Alexa Fluor ® 594 conjugated	1:800	IF	ThermoFisher Scientific, Waltham, MA, USA
Goat Anti-Rabbit IgG,<br>Alexa Fluor ® 488 conjugated	1:800	IF	ThermoFisher Scientific, Waltham, MA, USA

WB = Western Blot, IF = Immunofluorescence

Supplementary Figure S1

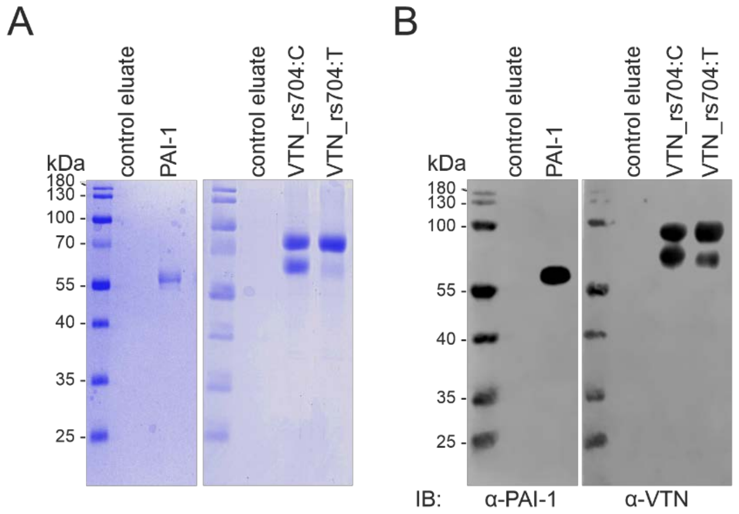

SUPPLEMENTARY FIGURE S1. Purification of Strep-tagged PAI-1, VTN_rs704:C and VTN_rs704:T. Purified recombinant proteins were subjected to SDS-PAGE and analysed *via* (A) Coomassie blue staining and (B) immunoblot (IB) analysis using antibodies against PAI-1 and vitronectin. Eluate obtained from cultivation medium of HEK293-EBNA transfected with empty pEXPR-IBA103 vector and subjected to the same purification procedure served as a control (control eluate).

Supplementary Figure S2

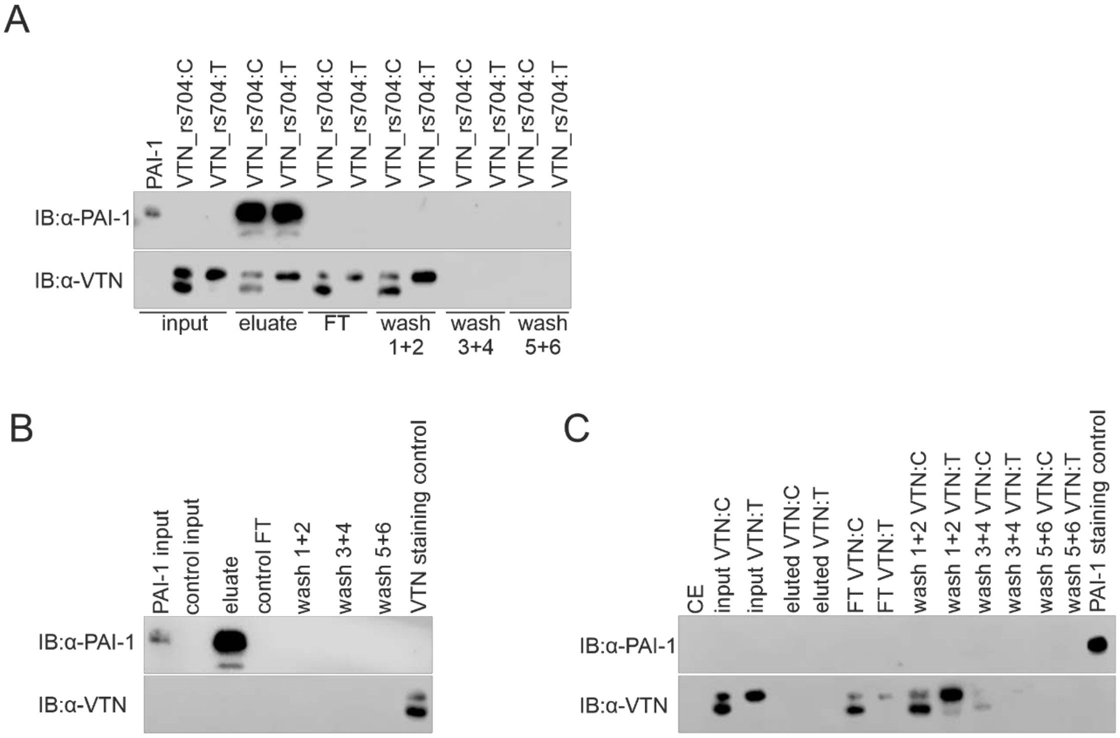

SUPPLEMENTARY FIGURE S2. Controls for the co-precipitation experiments. (A) Full-size western blots of Figure 2A, also revealing flow through (FT) and wash fractions (wash) prior to PAI-1 elution. (B) Vitronectin-free control input was loaded onto columns coupled with PAI-1 and subjected to the identical experimental procedure described for the vitronectin-containing input. After immunoblot (IB) analyses with antibodies against vitronectin, no staining was detected in the experimental fractions analysed. (C) Potential non-specific binding of vitronectin to the column material was tested by loading the vitronectin-containing input onto columns free of PAI-1 (coupled with empty control eluate; CE) and subjecting it to the same experimental procedure as described above. After IB analysis with antibodies against vitronectin and PAI-1, vitronectin was not detected in the elution fractions, but only in flow through and wash fractions, excluding non-specific binding of vitronectin to the column material.

Supplementary Figure S3

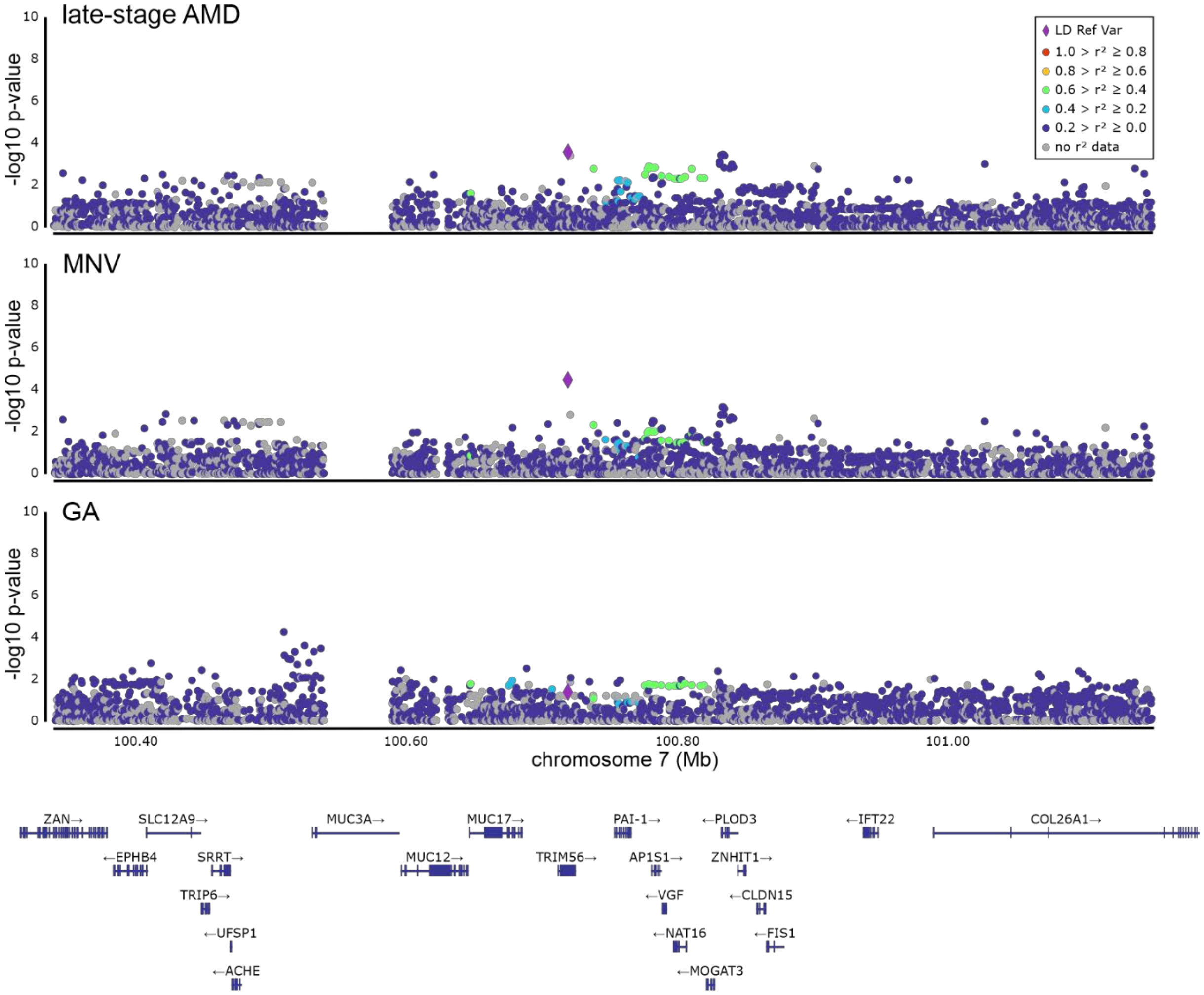

SUPPLEMENTARY FIGURE S3. Association of the genetic locus harboring the *PAI-1* gene with late-stage AMD phenotypes (all) and subgroups neovascular AMD (NV-AMD) and geographic atrophy (GA-AMD). The analysis within the IAMDGC dataset revealed only a minor association signal at this locus and low level of linkage disequilibrium (LD) among genetic variants in the selected intervals hampering association mapping. The variant with the smallest p-value, namely rs28709821, is presented as purple diamond and serves as linkage disequilibrium (LD) reference variant. Plots were generated with Locuszoom [26, 27]. A gene map with direction of transcription is shown at the bottom.

Supplementary Figure S4

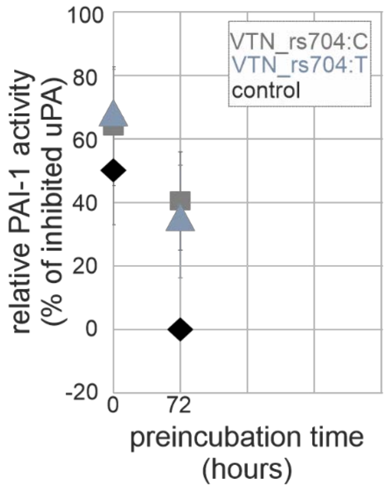

SUPPLEMENTARY FIGURE S4. PAI-1 activity assay after 0 and 72 hours preincubation of PAI-1 and vitronectin. VTN_rs704:C (grey squares), VTN_rs704:T (ice-blue triangles), or control eluate (control, black diamonds) were mixed with PAI-1 and incubated at 37°C. Aliquots of vitronectin/PAI-1 or control eluate/PAI-1 mixtures were subjected to the PAI-1 activity assay after 0 and 72 hours, measuring PAI-1-dependent uPA activity. The data were calibrated against the values obtained for control measurements at 72 hours (uPA at its maximum activity in our experimental setting, 100%). To better visualize the extent of PAI-1 inhibition and thus the inhibitory capacity of PAI-1, the calibrated uPA activity was subtracted from 100 % (maximal uPA activity). Data represent the mean ± SD of 3 replicates.

Supplementary Figure S5

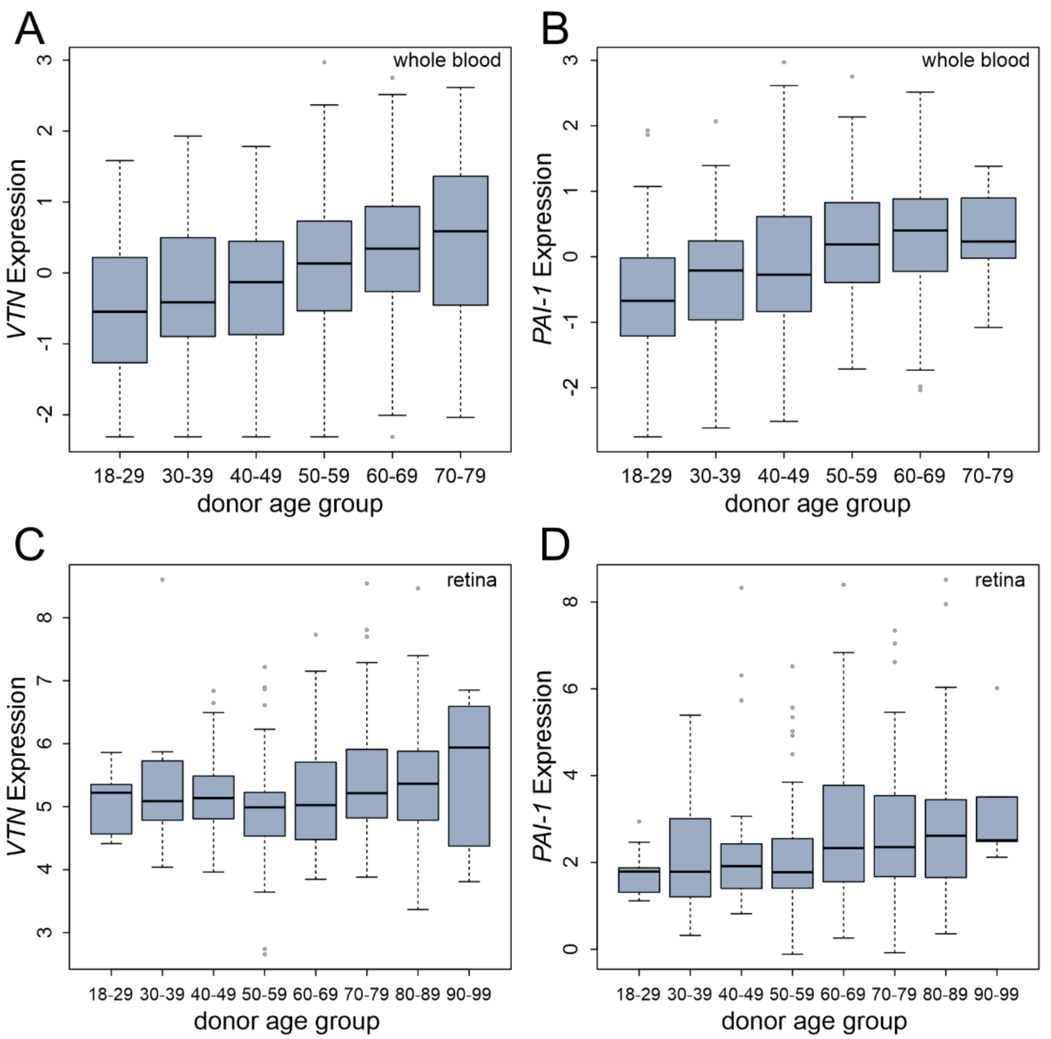

SUPPLEMENTARY FIGURE S5. Gene expression of *VTN* and *PAI-1* in human blood and retina separated by age groups. Donor individuals from whole blood samples (n = 556) [20] and healthy retinal tissues (n = 311) [22] were grouped according to their age at tissue donation. Shown are gene expression of (A) *VTN* and (B) *PAI-1* in whole blood and (C-D) in retinal tissue samples.
